# Chromosomes in the Porcine First Polar Body Possess Competence of Second Meiotic Division within Enucleated MII Stage Oocytes

**DOI:** 10.1371/journal.pone.0082766

**Published:** 2013-12-03

**Authors:** Tao Lin, Yun Fei Diao, Jung Won Kang, Jae Eun Lee, Dong Kyo Kim, Dong Il Jin

**Affiliations:** 1 Department of Animal Science & Biotechnology, Chungnam National University, Daejeon, Republic of Korea; 2 Department of Animal Biotechnology & Environment, National Institute of Animal Science, Rural Development Administration, Suwon, Republic of Korea; Institute of Zoology, Chinese Academy of Sciences, China

## Abstract

To determine whether chromosomes in the porcine first polar body (PB1) can complete the second meiotic division and subsequently undergo normal pre-implantation embryonic development, we examined the developmental competence of PB1 chromosomes injected into enucleated MII stage oocytes by nuclear transfer method (chromosome replacement group, CR group). After parthenogenetic activation (PA) or in vitro fertilization (IVF), the cleavage rate of reconstructed oocytes in the IVF group (CR-IVF group, 36.4 ± 3.2%) and PA group (CR-PA group, 50.8 ± 4.2%) were significantly lower than that of control groups in which normal MII oocytes were subjected to IVF (MII-IVF group, 75.8 ± 1.5%) and PA (MII-PA group, 86.9 ± 3.7%). Unfertilized rates was significantly higher in the CR-IVF group (48.6 ± 3.3%) than in the MII-IVF group (13.1 ± 3.4%). The blastocyst formation rate was 8.3 ± 1.9% in the CR-PA group, whereas no blastocyst formation was observed in the CR-IVF group. To produce tetraploid parthenogenetic embryos, intact MII stage oocytes injected with PB1chromosomes were electrically stimulated, treated with 7.5 μg/mL cytochalasin B for 3 h (MII oocyte + PB1 + CB group), and then cultured without cytochalasin B. The average cleavage rate of reconstructed oocytes was 72.5% (48 of 66), and the blastocyst formation rate was 18.7% (9 of 48). Chromosome analysis showed similar proportions of haploid and diploid cells in the control (normal MII oocytes) and CR groups after PA; overall, 23.6% of blastocysts were tetraploid in the MII oocyte + PB1 + CB group. These results demonstrate that chromosomes in PB1 can participate in normal pre-implantation embryonic development when injected into enucleated MII stage oocytes, and that tetraploid PA blastocysts are produced (although at a low proportion) when PB1 chromosomes are injected into intact MII stage oocytes.

## Introduction

The chromosomes in the first polar body (PB1) have the same genetic potential as their sister chromosomes remaining in the oocyte [1–5]. As human assisted reproductive technology (ART) has been limited by difficulties in acquiring human oocytes [6], it could be helpful to take full advantage of maternal genetic material derived from polar bodies.

Studies have shown that the polar bodies of mice are capable of participating in normal embryonic development in vitro and in vivo; producing normal offspring [2–5] or parthenogenetic embryonic stem (ES) cell lines [1]. It is difficult to extrapolate mouse results to large domestic animals because the latter require a much longer interval for oocyte development in vitro. However, results from domestic animals are far more relevant to the human condition than those from mice; in particular, the pig is regarded as the primary alternative species for xenotransplantation, due to their anatomical and physiological similarities to humans [7]. 

The tetraploid embryo complementation strategy has been widely used in animal reproduction. Tetraploid cells contribute to extraembryonic tissues, but rarely contribute to the embryo itself [8–10]. Chimeras produced with tetraploid embryos and ES cells, however, can produce genetically modified animals and may offer an alternative to somatic cell nuclear transfer. In domestic animals, however, ES cells contribute to chimera formation with extremely low efficiency [11], and it has proven very difficult to obtain tetraploid embryos from in vitro-produced porcine embryos, which showed a high frequency of polyspermic fertilization [12]. Thus, new strategies are needed to produce tetraploid parthenogenetic embryos in pigs. 

The first objective of this study was to determine whether porcine chromosomes in PB1 can participate in normal embryonic development. The second objective was to produce porcine tetraploid parthenogenetic embryos by injecting chromosomes of PB1 into intact MII oocytes. 

## Materials and Methods

### Chemicals

All of the chemicals used in our experiments were purchased from Sigma (St. Louis, MO, USA), except where indicated.

### Cumulus Oocyte Complexes (COCs) Collection and In Vitro Maturation

Porcine ovaries were obtained from a local slaughterhouse (NH Livestock Cooperation Association, Nonsan City, Chungnam Province, Korea) where we had acquired permission to use porcine ovaries, and transported to the laboratory within 2 h in physiological saline at 30 to 35°C. Follicles of 3 to 6 mm in diameter were aspirated from the ovarian surface using an 18-gauge needle attached to a 10-ml disposable syringe. Oocytes with a uniform ooplasm and compact cumulus cell mass were selected for in vitro maturation. These COCs were cultured in 500 μl TCM medium supplemented with 10% porcine follicular fluid (PFF), 10 ng/ml epidermal growth factor (EGF), 10 IU/ml PMSG and 10 IU/ml hCG in each well of a four-well multi dish. After culture for 22 h, the COCs were transferred to the same medium without PMSG or hCG, and cultured for another 22 h. 

### Evaluation and collection of porcine PB1s

**Figure 1:**
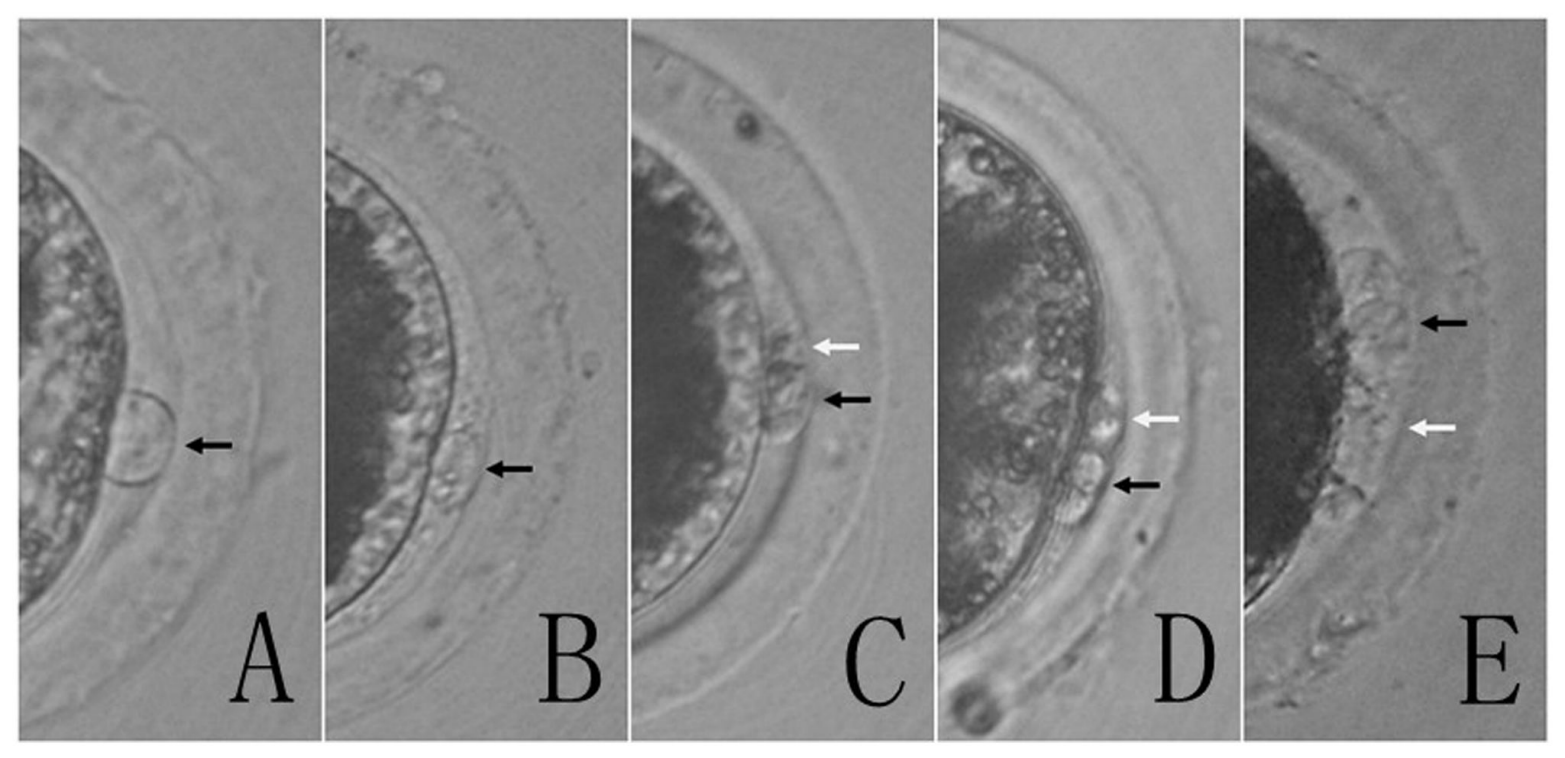
Morphological analysis identifies five categories of porcine first polar bodies. Grade 1: Round or ovoid PB1 with intact smooth membrane (A). Grade 2: Round or ovoid PB1 with intact membrane (B). Grade 3: Broken PB1 with a small PB1 fragment (C). Grade 4: Broken PB1 with a big PB1 fragment (D). Grade 5: Broken PB1 completely (E). Grade 1, 2 were considered as good quality. Black arrows: Polar bodies. White arrows: Fragments.

**Figure 2:**
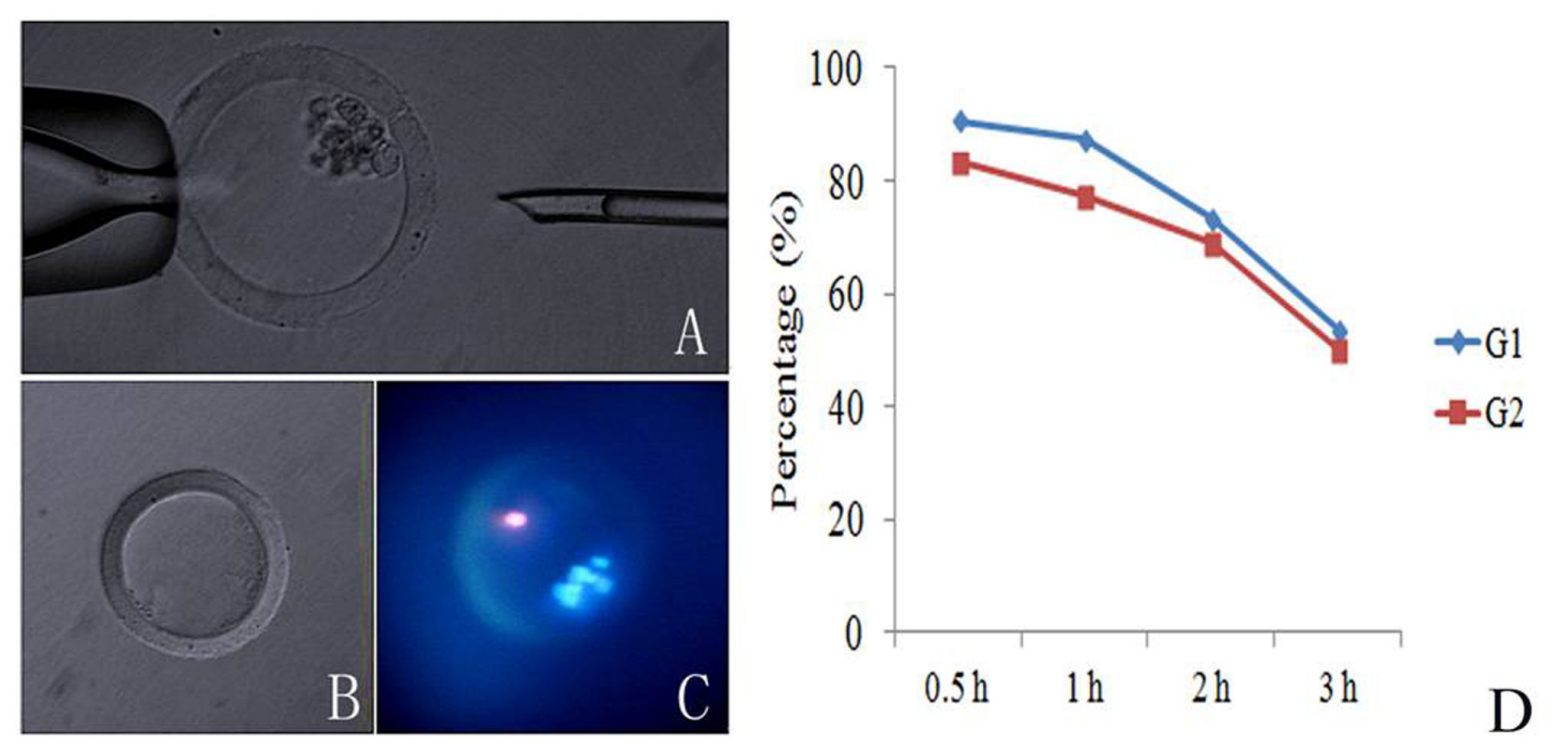
Collection and viability identification of the PB1s. A: PB1 with intact membrane was packed into an empty zona pellucida using a fine glass needle. B: An empty zona pellucida. C: The viability of the PB1s was evaluated by propidium iodide (red, dead) and Hoechst 33342 (blue, both living and dead) staining. D: Percentages of PB1 at various times after being packed into empty zona pellucida. G1 and G2 stand for Grade 1 and Grade 2 PB1s, respectively.

Following in vitro maturation, cumulus cells were removed by treatment with 0.1% hyaluronidase in HEPES-buffered Tyrode’s medium (TLH) containing 0.1% (wt/vol) polyvinyl alcohol (TLH-PVA). Oocytes with PB1 were selected, placed in manipulation medium (DPBS plus 20% FBS) containing 5 μg/ml cytochalasin B, and overlain with mineral oil. Finally, PB1s were evaluated and collected by methods previously described with a few modifications [5,13]. Briefly, five categories of PB1s were investigated according to their morphology under an inverted microscope. PB1s (round or ovoid) with intact membrane (smooth surface or not) were considered as good quality (Figure 1). In order to collect PB1s, an empty zona pellucid was prepared from random oocyte. Porcine PB1s with intact membranes were grouped (5-7/group) packed into an empty zona pellucid using a fine glass needle. The viability of PB1s was evaluated by propidum iodide and Hoescht 33342 dual staining (Figure 2).

### Injection of PB1 chromosomes into enucleated or intact MII oocytes

**Figure 3:**
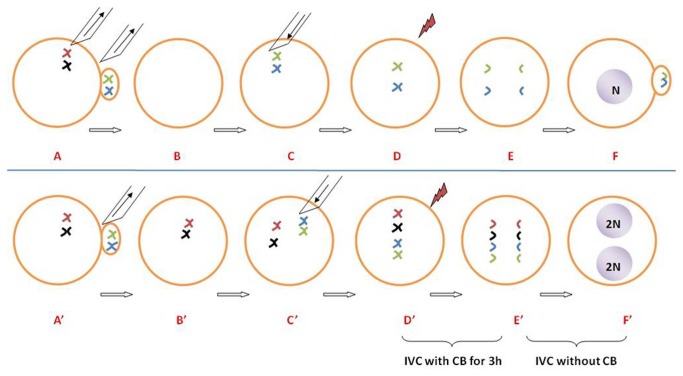
Schematic of our strategy for using PB1 chromosomes to produce haploid (A-F) and tetraploid (A’-F’) parthenogenetic embryos. A: Removal of nuclear materials from PB1 and MII oocytes. B: Enucleated MII oocyte. C: Injection of PB1 chromosomes into an enucleated MII oocyte. D: Electrical stimulation. E: Segregation of sister chromatids. F: The polar body is extruded and the embryo becomes haploid. A’: Removal of the first polar body from an MII oocyte. B’: MII oocyte with nuclear materials and without PB1. C’: PB1 chromosomes are injected into the intact MII oocyte. D’: Electrical stimulation. E’: Culture of the reconstructed oocytes with cytochalasin B (3 h, to inhibit polar body ejection). F’: The tetraploid embryo.

The plasma membrane of the PB1 was broken by aspiration with a glass needle, and the nuclear materials of the PB1 were directly injected into cytoplasm of enucleated or intact MII oocytes (Figure 3 A-C, A’-C’). 

### Activation and in vitro culture of reconstructed oocytes

The present study examined two type of reconstructed oocytes: 1) enucleated MII stage oocytes injected with PB1 chromosomes (chromosome replacement group, CR group); and 2) intact MII stage oocytes injected with PB1 chromosomes (MII oocyte + PB1 group). Briefly, reconstructed oocytes were washed three times with activation solution (0.3 M D-mannitol, 0.1 mM MgSO_4_, 0.05 mM CaCl_2_, 0.01% PVA) and activation was induced with a direct current-pulse of 1.5 kV/cm for 100 μs, using an Electro cell manipulator 2001 (BTX, Inc., San Diego, CA, USA). After each activation treatment, reconstructed oocytes from the CR group, were washed with PZM-3 containing 3 mg/ml bovine serum albumin (BSA), transferred into 50-μl microdrops of the same culture media, covered with mineral oil in a polystyrene culture dish, and incubated at 38.5°C for 7 days under an atmosphere of 5% CO_2_. Reconstructed oocytes from the MII oocyte + PB1 group were cultured in PZM-3 containing 3 mg/ml BSA and 7.5 μg/ml cytochalasin B to inhibit extrusion of the polar body. After activation for 3 h, reconstructed oocytes were transfer to PZM-3 without cytochalasin B. In MII stage oocytes and enucleated oocytes groups, the methods of activation and in vitro culture are the same as CR group. The day of PA was designated as day 1, and cleavage and blastocyst formation were assessed at 3 day and 7 day, respectively.

### In vitro fertilization and embryo culture

Reconstructed oocytes from the CR group and MII stage oocytes were subjected to in vitro fertilization (IVF), as described by Lee et al [14]. Briefly, CR oocytes and MII stage oocytes were washed three times in mTBM (modified Tris-buffered medium; 113.1 mM NaCl, 3 mM KCl, 7.5 mM CaCl_2_, 11 mM glucose, 20 mM Tris, 2 mM caffeine, 5 mM sodium pyruvate, and 2 mg/ml BSA). For fertilization, groups of 10-12 oocytes were transferred into 45-μl droplets of mTBM and covered with mineral oil. A fresh semen sample was washed three times by centrifugation with DPBS supplemented with 0.1% BSA at 2000 g for 3 min. After washing, the sperm pellet was resuspended to 1×10^6^ cells/ml with mTBM, and 5 μl was added to each 45-μl CR-oocyte-containing mTBM droplet. The droplets were incubated for 5-6 h at 38.5°C in a humidified atmosphere of 5% CO_2_. The resulting gametes were washed three times and cultured in 50 μl of PZM-3. The day of IVF designated as day 1, and cleavage and blastocyst formation were evaluated on day 3 and day 7, respectively.

### Nuclear staining

Parthenogenetic blastocysts were washed with PBS containing 0.1% polyvinylpyrrolidone (PVP), and fixed with 4% paraformaldehyde. Nuclei were stained with DAPI for 5 min at room temperature, and the blastocysts were mounted on glass slides and squashed gently with a cover slip.

### Karyotype analysis

Chromosome analysis was performed with a modification of the procedure described by Hao et al [15]. Briefly, on day 6 of culture, 0.2 μg/ml colcemid was added to the PZM-3, and the embryos were cultured for an additional 6 h. The zona pellucida was removed by treatment with 5 mg/ml pronase, and the embryos were placed in 0.8% sodium citrate (wt/vol) for 3 min, and then in 75 mM KCl for 2 min. Blastocysts were transitory fixed in fixing medium (methanol:acetic acid = 3:1 [v/v]), transferred onto slides, and overlain with methanol:acetic acid (1:1 [v/v]). Each slide was then air-dried, stained with DAPI, and mounted with a coverslip.

### Assessment of fertilization parameters

Sperm penetration and pronuclear formation was assessed following 10-12h after IVF. The zona pellucida of the oocytes was removed with 0.25% pronase, the zona-free embryos were fixed in 4% (wt/vol) paraformaldehyde in PBS for 15 min. The fixed embryos were then stained with DAPI in VECTASHIELD^®^ mounting medium (Vector Laboratories, Inc. Burlingame, CA 94010). Embryos were next mounted with slight coverslip compression and evidence of sperm penetration and pronuclear formation were observed under an epifluorescence microscope. The presence of two pronuclei was designated as monospermic fertilization, and three or more pronuclei was taken as polyspermic fertilization. Other cases were considered as unfertilized.

### Statistical analysis

At least three replicates were performed for each test, and statistical analyses were carried out using SPSS 17.0 (SPSS Inc., Chicago, IL, USA). All percentage data were analyzed by one-way ANOVA, followed by Duncan’s multiple range test. The results are expressed as means ± SE. Percent data were transformed into arcsine prior to statistical analysis P < 0.05 was considered to be statistically significant. 

## Results

### Morphology and viability of the PB1

**Figure 4:**
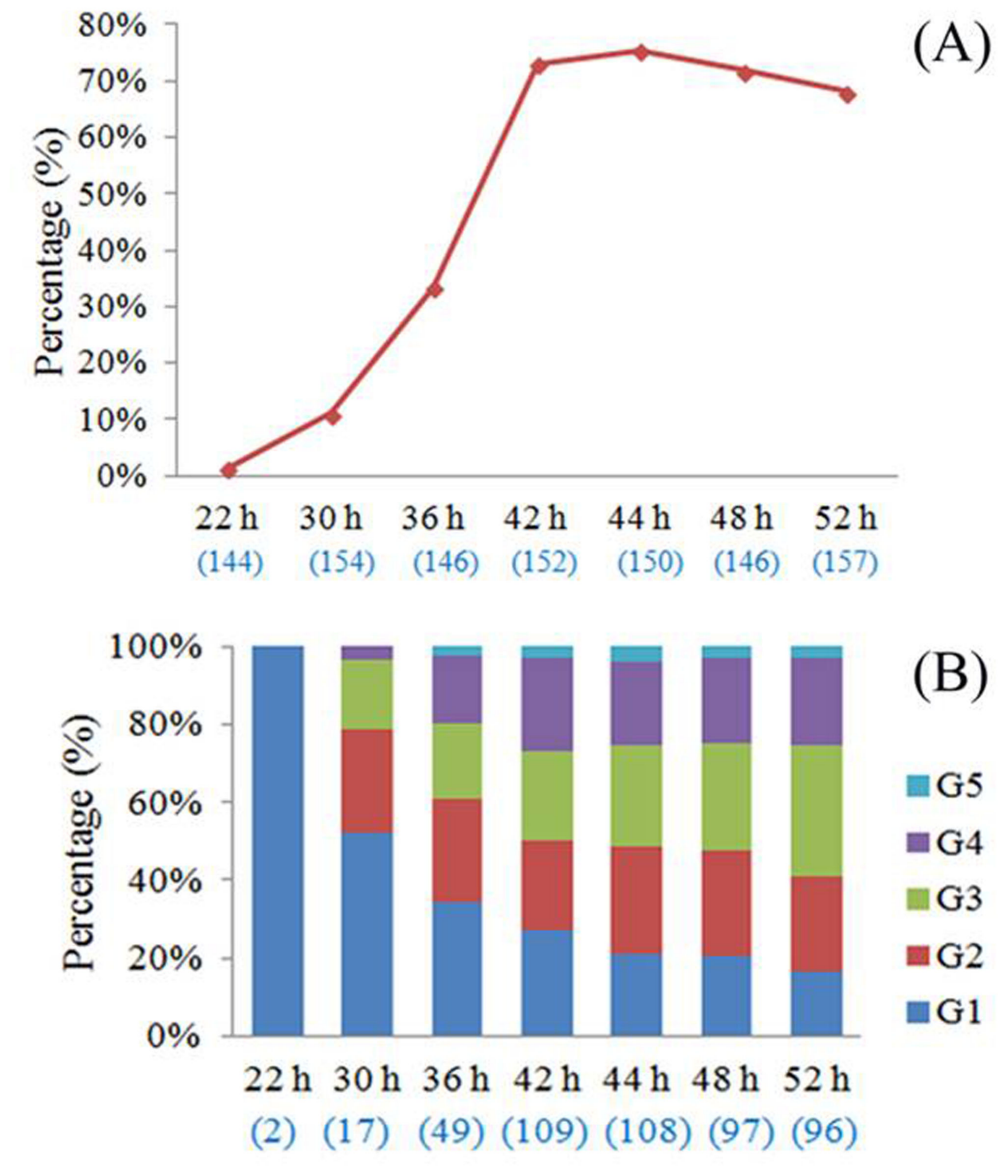
Extrusion rate (A) and morphology (B) of PB1s. The number of oocytes examined in each group is given in parentheses. G1, G2, G3, G4, and G5 represent Grade 1, Grade 2, Grade 3, Grade 4, and Grade 5, respectively.

The extrusion rates and morphological categories for the PB1s were observed after maturation for 22, 30, 36, 42, 44, 48, 52 h. As shown in Figure 4, the PB1 extrusion rate clearly increased with in vitro maturation time, peaking at 42 and 44 h (Figure 4 A). However, the proportion of good quality PB1s (Grade 1 or 2) decreased with in vitro maturation time (Figure 4 B). Percentages of live PB1s were evaluated by propidium iodide and Hoechst 33342 dual staining at various times (Figure 2 A-C). Grade 1 and 2 PB1s maintained higher proportions of viability at 1 h (more than 83% and 75%, respectively), but this viability decreased as the in vitro maturation time increased (Figure 2 D).

### In Vitro Development of the CR Group

**Figure 5:**
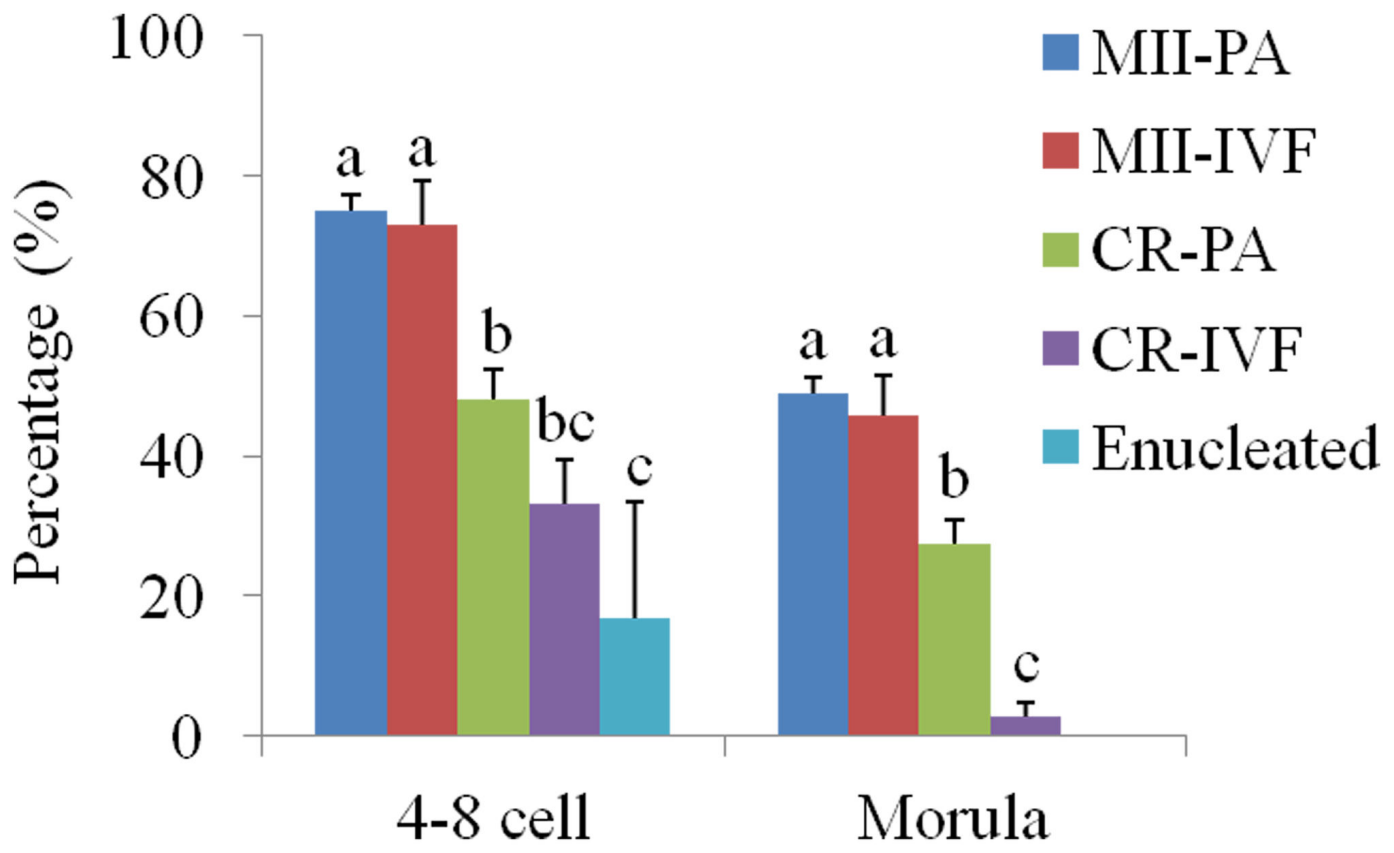
In vitro development (4-8 cell or morula) of reconstructed embryos after PA and IVF (also see Table 1). Rates are given as the percentage of 4-8 cell or morula embryos versus total cleavages. The different letters above the bars indicated statistically significant differences (p < 0.05).

As shown in Table 1, after PA and IVF, the percentage of recombined oocytes that underwent cleavage in the CR-IVF group (36.4 ± 3.2%) and CR-PA group (50.8 ± 4.2%) were significantly lower than that of control groups in which MII-IVF group (75.8 ± 1.5%) and MII-PA group (86.9 ± 3.7%), but significantly higher than that in the enucleated oocytes group (7.9 ± 1.9%). There was no significant difference between the CR-PA and CR-IVF groups at the 4-8 cell stage, but morula formation rate was significantly higher in CR-PA group than in CR-IVF group (Figure 5). Furthermore, the blastocyst formation rate was 8.3 ± 1.9% in the CR-PA group, but no blastocyst formation was observed in the CR-IVF group (Table 1). There was no significant difference in the total cell number of blastocysts between CR-PA and MII-PA groups (23.2 ± 2.6 and 31.1 ± 1.9, respectively).

**Table 1:** In vitro development of porcine reconstructed embryos from enucleated MII oocytes injected with chromosomes from the first polar body.

Group	Method of activation	Oocytes examined	% Of oocytes developed		Cell number of blastocysts
			≥ 2 Cell	Blastocyst	
MII oocyte	PA	165	145 (86.9 ± 3.7)^a^	29 (20.2 ± 1.2)^a^	31.1 ± 1.9^ab^
MII oocyte	IVF	190	143 (75.8 ± 1.5)^a^	25 (18.1 ± 1.9)^a^	35.8 ± 2.6^a^
Chromosome replacement (CR)	PA	129	66 (50.8 ± 4.2)^b^	5 (8.3 ± 1.9)^b^	23.2 ± 2.6^b^
Chromosome replacement (CR)	IVF	186	67 (36.4 ± 3.2)^c^	0 (0.0 ± 0.0)^c^	0.0 ± 0.0^c^
Enucleated oocyte	PA	65	5 (7.9 ± 1.9)^d^	0 (0.0 ± 0.0)^c^	0.0 ± 0.0^c^

Data with different superscripts are significantly different (p < 0.05).

### In vitro development of the MII oocyte + PB1 group

PB1 chromosomes were injected into intact MII stage oocytes, electrically stimulated, treated with 7.5 μg/mL cytochalasin B for 3 h, and then cultured without CB. The majority (72.5%, 48 of 66) of the reconstructed oocytes showed normal morphology and cleavage, and we observed a blastocyst formation rate of 18.7% (9 of 48). DAPI staining revealed that the average cell number in the blastocysts was 22.3 ± 2.0.

### Ploidy of porcine parthenogenetic embryos

**Figure 6:**
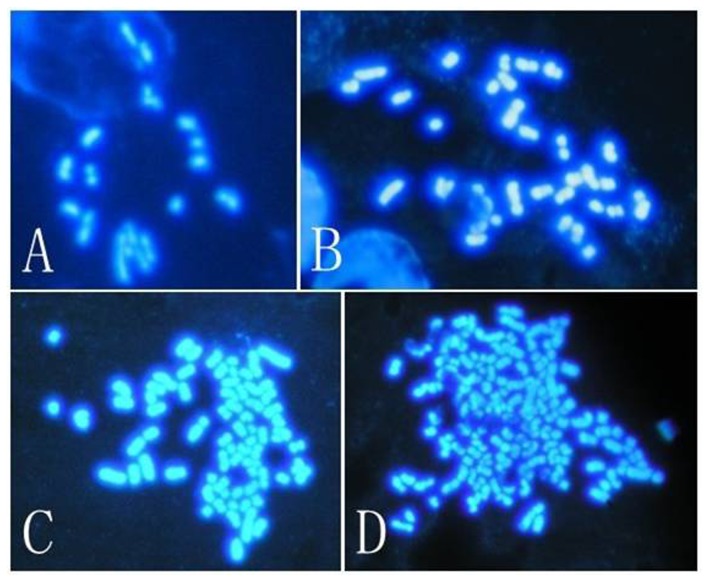
Karyotype analysis of day-7 PA blastocysts. A: Haploid (n = 19). B: Diploid (n = 38). C: Tetraploid (n = 76). D: Polyploid (n = 152). Magnification: × 1000.

The chromosomes of the reconstructed parthenogenetic blastocysts were analyzed (Table 2, Figure 6). For control embryos, 55.6%, 28.6% and 15.9% were found to be haploid, diploid and mosaic/polyploid, respectively. Similar proportions were found in the CR group (41.7%, 35.4%, 22.9%, respectively), whereas in the MII oocyte + PB1 + CB group, 23.6% (7 of 29) of the blastocysts were tetraploid, 12.8% were haploid, 36.5% were diploid, and 27.1% were mosaic or polyploid.

**Table 2:** Ploidy of porcine parthenogenetic reconstructed embryos produced by the different methods.

Group	Oocytes examined	HaploidN	Diploid2N	Tetraploid4N	Mosaic or polyploid*
MII oocyte	26	14 (55.6%)	8 (28.6%)	0 (0%)	4 (15.9%)
CR	12	5 (41.7%)	4 (35.4%)	0 (0%)	3 (22.9%)
MII oocyte + PB1 + CB	29	4 (12.8%)	10 (36.5%)	7 (23.6%)	8 (27.1%)

^*^ Mosaic, 1N/2N or 2N/4N; and polyploid, > 4N.

### Fertilization parameter after IVF

In Table 3, it was shown that the unfertilized rate was significantly higher in the CR-IVF group (48.6 ± 3.3%) than in the control (13.1 ± 3.4%), and monospermy and polyspermy rates were significantly lower in CR-IVF group (14.5 ± 3.3%, 36.9 ± 3.0%, respectively) than in the control group (29.1 ± 1.8%, 57.8 ± 2.9%, respectively).

**Table 3:** Fertilization parameter of porcine reconstructed embryos from enucleated MII oocytes injected with chromosomes from the first polar body after IVF.

Parameter	Experiment groups	
	CR-IVF	MII-IVF
No. of oocytes examined	73	102
Unfertilized (%)	48.6 ± 3.3^a^	13.1 ± 3.4^b^
Monospermy (%)	14.5 ± 3.3^a^	29.1 ± 1.8^b^
Polyspermy (%)	36.9 ± 3.0^a^	57.8 ± 2.9^b^

With the same row, different superscripts indicate significant differences (p < 0.05).

## Discussion

In mice, oocytes reconstructed with live polar bodies developed normally and produced offspring following injection of spermatozoon [4,5]. In pigs, nuclear materials from fresh or frozen-thawed live porcine first polar bodies also supported oocyte fertilization and subsequent embryonic development [16]. These studies showed that chromosomes from live (but not dead) polar bodies can participate in normal embryonic development in vitro and/or in vivo. In our laboratory, we obtained similar results, with porcine chromosomes from dead polar bodies failing to support embryonic development (data not shown). 

In the present study, we investigated the quality and proportion of porcine live polar bodies. First, we assessed the morphology and viability of the obtained PB1s. We found that the porcine PB1 extrusion rate increased with the in vitro maturation time, peaking at 42 and 44 h. However, the proportions of oocytes with good quality (Grades 1 or 2) PB1s showed a decreasing trend over time (Figure 4). To evaluate viability of the PB1s, we used dual staining with propidium iodide (red, dead cells) and Hoechst 33342 (blue, live and dead cells). The Grade 1 and 2 PB1s had higher proportions of viability at 1 h, but these proportions decreased over time. To obtain fresh and viable PB1s, we therefore collected PB1s after 38 to 40 h of IVM.

Two polar bodies (the first and second polar bodies) are extruded from the body of a large main oocyte during meiotic division in mammals. Within the polar bodies, the chromosomes degenerate quickly and do not participate in subsequent embryonic development [4]. Polar bodies have been analyzed for pre-implantation genetic screening, to avoid the fertilization and transfer of aneuploid oocytes during human IVF [17]. Only a few reports have indicated that polar bodies can also be developed into embryos [2,4,5,16].

In the present study, the reconstructed oocytes of CR-PA group cleaved normally and developed into blastocysts after PA, although cleavage and blastocyst formation rates were significantly lower than control (MII-PA group), indicating that MII cytoplasm allowed the chromosomes of the PB1 to complete the second meiotic division and participate in subsequent pre-implantation embryonic development. In contrast, relatively few (7.9 ± 1.9%) enucleated oocytes without PB1 chromosomes showed successful cleavage after electrical stimulation. Therefore, we speculate that the CR-PA groups could have included some cases of incomplete enucleation (in our preliminary trials, we found that the incomplete enucleation rate was less than 5%, data not shown) or cytoplasm fragmentation after PA, but not at high proportions. Although, no blastocyst formation was observed when reconstructed oocytes of CR group were subjected to IVF, these oocytes can cleave normally and develop to > 8-cell embryos and/or morula (Figure 5). To further ensure whether no blastocyst formation might be due to fertilization failure, we observed fertilization parameter of porcine reconstructed embryos from enucleated MII oocytes injected with chromosomes from PB1 after IVF. As expected, unfertilized rate was significantly higher in the CR-IVF group compared with the control group (Table 3). Thus, we speculated that abnormal integration of injected PB1 chromosomes in enucleating MII stage oocytes should have led to decrease efficiency of fertilization and fail to develop to blastocysts.

A previous study showed that fresh or frozen-thawed porcine PB1s injected into enucleated oocytes and subjected to intracytoplasmic sperm injection (ICSI) developed beyond the 8-16 cell stage [16]. In mice, in contrast, normal living pups were produced from zygotes reconstructed with fresh first or second polar bodies, or frozen second polar bodies [2,4,5]. Thus, there are differences between the results from polar-body-derived mouse recombinant oocytes and those of the pig, whose oocytes are considered to be particularly fragile. In most cases, tetraploid embryos have been obtained by electric fusion of diploid blastomeres or inhibition of cell division by cytochalasin B treatment of fertilized oocytes [18–21]. In pigs, however, it is very hard to produce tetraploid embryos from fertilized oocytes, due to high rates of polyspermic fertilization. A recent study found that porcine tetraploid parthenogenetic embryos could be produced using cytochalasin B, which inhibited the extrusions of the first and second polar bodies [12]. 

In the present study, we report a new method of producing porcine tetraploid parthenogenetic embryos (Figure 3) by injecting PB1 chromosomes into intact MII stage oocytes, and then subjecting them to electrical stimulation and treatment with cytochalasin B (to inhibit extrusion of the polar body). Although the cleavage rates were similar after PA, the blastocyst formation rates were significantly lower in the MII oocyte + PB1 + CB group compared to the normal MII oocyte group (data not shown). Interestingly, the cleavage and blastocyst formation rates were higher in the MII oocyte + PB1 + CB group compared to the RC-PA group (72.5% and 18.7% versus 50.8% and 8.3%, respectively). We speculate that intact MII stage oocytes may support the ability of PB1 chromosomes to participate in embryonic development more efficiently than enucleated MII cytoplasm.

We observed similar karyotypes between the CR group and normal MII oocyte groups after PA. This supports the notion that PB1 chromosomes have the same genetic potential as their sister chromosomes remaining in the oocyte [4]. Reconstructed parthenogenetic embryos from the MII oocyte + PB1 + CB group included 23.6% (7 of 29) tetraploid embryos. Although this proportion was relatively low, our findings confirm that chromosomes within the first polar bodies can participate in embryonic development in intact MII stage oocytes. 

### Perspectives

Porcine oocytes are sensitive to low temperature. Limited survival is seen after thawing, and no study to date has reported the production of live offspring following transfer of vitrified porcine oocytes. One major obstacle limiting the cryopreservation of oocytes is their large proportion of cytoplasmic lipids [22–24]. To reduce intracellular lipid contents, researchers have developed methods for centrifugation and partial removal of lipid droplets [25,26]. However, the polar body is a small cellular body that contains less cytoplasm, and thus could have a higher tolerance for the cooling. Polar bodies are believed to contain the same genetic material as the oocyte, so successful cryopreservation of polar bodies could be a viable method for preserving nuclear materials from female pigs. The single study performed to date using porcine polar bodies did not yield promising results [16], so further studies are warranted. A lack of oocytes is a key problem in human ART. However, oogenesis leads to the formation of one oocyte and three polar bodies. Since studies have shown that polar bodies can be used to create normal offspring [4], it could be theoretically possible to reproduce four offspring using the chromosomes from one oocyte. At present, the biological functions of the polar bodies in large domestic animals are poorly understood [16], and additional work is needed. Although experimental animal studies do not precisely emulate human IVF procedures, our novel data provide important insights that may facilitate the future development of human Assisted Reproductive Technologies. 

## Conclusions

We herein show for the first time that porcine chromosomes in the PB1 can undergo the second meiotic division within enucleated MII stage oocytes, and (following PA) can cleave normally and develop to the blastocyst stage. However, there was no blastocyst formation after IVF. We also herein report that tetraploid PA blastocysts were successfully produced (albeit at a relatively low rate) when PB1 chromosomes were injected into intact MII stage oocytes.
